# Neuropeptide Y Is Produced by Adipose Tissue Macrophages and Regulates Obesity-Induced Inflammation

**DOI:** 10.1371/journal.pone.0057929

**Published:** 2013-03-05

**Authors:** Kanakadurga Singer, David L. Morris, Kelsie E. Oatmen, Tianyi Wang, Jennifer DelProposto, Taleen Mergian, Kae Won Cho, Carey N. Lumeng

**Affiliations:** 1 Department of Pediatrics and Communicable Diseases, University of Michigan Medical School, Ann Arbor, Michigan, United States of America; 2 Department of Molecular and Integrative Physiology, University of Michigan Medical School, Ann Arbor, Michigan, United States of America; Laboratory of Neuroendocrine-Immunology, Pennington Biomedical Research Center, United States of America

## Abstract

Neuropeptide Y (NPY) is induced in peripheral tissues such as adipose tissue with obesity. The mechanism and function of NPY induction in fat are unclear. Given the evidence that NPY can modulate inflammation, we examined the hypothesis that NPY regulates the function of adipose tissue macrophages (ATMs) in response to dietary obesity in mice. NPY was induced by dietary obesity in the stromal vascular cells of visceral fat depots from mice. Surprisingly, the induction of *Npy* was limited to purified ATMs from obese mice. Significant basal production of NPY was observed in cultured bone marrow derived macrophage and dendritic cells (DCs) and was increased with LPS stimulation. *In vitro*, addition of NPY to myeloid cells had minimal effects on their activation profiles. NPY receptor inhibition promoted DC maturation and the production of IL-6 and TNFα suggesting an anti-inflammatory function for NPY signaling in DCs. Consistent with this, NPY injection into lean mice decreased the quantity of M1-like CD11c^+^ ATMs and suppressed Ly6c^hi^ monocytes. BM chimeras generated from *Npy^−/−^* donors demonstrated that hematopoietic NPY contributes to the obesity-induced induction of *Npy* in fat. In addition, loss of *Npy* expression from hematopoietic cells led to an increase in CD11c^+^ ATMs in visceral fat with high fat diet feeding. Overall, our studies suggest that NPY is produced by a range of myeloid cells and that obesity activates the production of NPY in adipose tissue macrophages with autocrine and paracrine effects.

## Introduction

Obesity correlates with the induction of a chronic state of inflammation that is linked to metabolic dysfunction [1]. Clinical studies demonstrate elevations in c-reactive protein (CRP) and pro-inflammatory cytokines in obese children and adults [2], [3]. A key event in obesity-induced inflammation is the alteration in the inflammatory profile of adipose tissue macrophages (ATMs) [4], [5]. In the lean state, adipose tissue contains a population of resident macrophages which express markers of alternative activation (M2). With obesity, there is a shift in the features of the ATM population towards a classical activation (M1) profile driven by the appearance of CD11c^+^ ATMs. The induction of CD11c^+^ ATMs appears to be closely linked to the increase in circulating Ly6c^hi^ monocytes seen with dietary obesity [6]. Compared to Ly6c^lo^ monocytes, Ly6c^hi^ monocytes are preferentially recruited to active areas of inflammation including atherosclerotic plaques [7] and in obesity are thought to drive M1 ATM accumulation in adipose tissue [6].

There are a wide range of triggers for adipose tissue inflammation that occur in the setting of diet induced obesity and increased adiposity. These include lipolytic signals, chemokines, chemoattractants, cytokines, and pathogen associated molecular proteins (PAMPs) such as lipopolysaccharide [4], [8], [9]. These signals activate a range of pro-inflammatory signaling pathways that include chemokine receptors (e.g. CCR2) and a host of innate immune pattern recognition receptors as Toll-like receptors (TLRs), Nod-like receptors (NLRs) and C-type lectin receptors (CLRs). Attenuation of many of these inflammatory signals has been shown to improve metabolic disease [10], [11]. Hence, identification of the signals that trigger adipose tissue inflammation is critical. Physiologic stress signals have been shown to be activated with obesity and induce inflammation in adipose tissue [12], [13]. While there are many neurohumoral factors associated with stress responses, neuropeptide Y (NPY) is a dominant hormone that is elevated in chronic stress and sympathetic nervous system activation [13].

NPY is a peptide hormone that binds to a family of 5 G-protein coupled receptors (Y1– Y5) found in multiple tissues [14]. NPY receptors are found in a broad array of tissues including those involved in metabolism such as adipose and liver. In immune cells, only Y1, Y2, and Y5 receptors have been identified [15]. The best characterized function of NPY in obesity is in the CNS where neuronal NPY stimulates orexigenic pathways via Y1 receptor activation [16]. However, there is evidence that NPY influences metabolic function in peripheral tissues as well, mostly via Y2 and Y5 receptors although Y1 receptors have been studied on adipocytes themselves. For example, with obesity, NPY is induced in adipose tissue where it may regulate multiple aspects of adipocyte biology. In adipocytes, NPY decreases lipolysis and promotes adipogenesis [17], [18] suggesting that NPY has beneficial effects on lipid uptake and storage in fat. Consistent with this, NPY deficient mice are more obese and insulin resistant with high fat diet feeding [19]. However, in a model combining chronic stress and diet-induced obesity, NPY induction in fat correlated with insulin resistance and an increase in ATMs [20], [21]. In this model, blockade of the NPY 2 receptor (Y2R) decreased ATM accumulation and insulin resistance in stress-induced obesity models [20]. The source of NPY and the pattern of NPY receptor activation in obese adipose tissue are unknown.

Relevant to ATM biology is the evidence that NPY is a potent immune mediator with both pro-inflammatory and anti-inflammatory actions on leukocytes. Exogenous NPY protects against experimental autoimmune encephalitis and decreases inflammation in experimental sepsis [22], [23]. NPY can also decrease macrophage oxidative burst via Y1, Y2, and Y5 NPY receptors [15] and decrease neutrophil and T-lymphocyte tissue infiltration [24], [25]. Overall, the effects of NPY on leukocytes may be highly context dependent as Y1 activation has been shown to downregulate T cell responses, but paradoxically activate antigen presenting cells [26].

Given that NPY has a role in immune cell activity and obesity, we investigated the hypothesis that NPY influences the activity of ATMs as a potential link between stress signals, obesity, and inflammation. This investigation led to us to find evidence that NPY is produced by macrophages in visceral fat and that ATMs are the primary source of the increase in *Npy* expression seen in fat with obesity. NPY production and inflammatory regulation in myeloid cells was confirmed in bone marrow (BM) derived cells. *In vitro* studies and BM chimeras suggest that myeloid cell derived NPY has anti-inflammatory autocrine and paracrine effects within adipose tissue.

## Methods

### Animals and Animal Care

Mice used in these experiments were male C57Bl/6J, 129S-NPY^tm1Rpa^/J (NPY-KO), and control 129S mice (Jackson laboratories). Mice were fed *ad lib* either a control normal diet (ND) consisting of 4.5% fat (5001;LabDiet) or a high fat diet (HFD) of 60% of calories from fat (Research Diets, Inc., D12492) starting at 6–8 weeks of age for 16 weeks unless specified. Animals were housed in a specific pathogen-free facility with a 12 h light/12 h dark cycle and given free access to food and water. All animal use was in compliance with the Institute of Laboratory Animal Research Guide for the Care and Use of Laboratory Animals and approved by the University Committee on Use and Care of Animals at the University of Michigan (Animal welfare assurance number A3114-01). Mice were injected I.P. with NPY scrambled peptide (Tocris 3903, 60 µg/kg/day) or NPY recombinant peptide (Tocris 1153, 60 µg/kg) daily for 5 or 10 days (n = 5 per group) based on prior reports in rodents [27].

### Real-time RT-PCR

RNA extraction was performed with an RNeasy kit (Qiagen). RT reactions were performed and real-time PCR analysis was performed normalized to GAPDH (SYBR Green, ABI Prism 7200 Sequence Detection System; Applied Biosystems). Relative expression was assessed by the comparative C_T_ method correcting for amplification efficiency of the primers and performed in duplicate. PCR primers used are reported in Table S1.

### Adipose Tissue Stromal Vascular Fraction (SVF) Isolation and Flow Cytometry

Adipose tissue fractionation and flow cytometry analysis was performed as described [28]. SVF cells were stained with F4/80-APC, CD11b-APC-Cy7, and CD11c-PE-Cy7 (eBioscience) along with propidium iodide for viability [29]. Fluorescent activated cell sorting (FACS) of PI^−^ ATMs (F4/80^+^ CD11b^+^) and non-ATMs (F4/80^−^ CD11b^−^) cells was performed at University of Michigan flow cytometry core (BD FACS Aria).

### Cell Culture

Bone marrow cells were isolated from C57Bl/6 mice by flushing of tibia and fibula. After RBC lysis cells were plated at 1.5×10^6^cells/ml. Cells were differentiated into bone marrow derived macrophages (BMMP, L929 conditioned media) or bone marrow derived dendritic cells (BMDC, GM-CSF) for 6 days. Differentiation was confirmed by demonstrating F4/80 expression in BMMP and CD11c and MHCII expression in BMDC by flow cytometry. Cells were then placed in 10% serum media for 24 hours prior to treatment with NPY (100 uM), LPS (10–100 ng/ml), IL-4 (20 ng/ml) for 18–24 hours. 3T3-L1 cells were differentiated into adipocytes as previously described [4]. NPY ELISA studies were performed on media with the use of NPY high sensitivity kit from Bachem (S-1145).

### Hepatic Triglyceride Content

Livers were weighed, snap frozen in liquid nitrogen, and stored at -80 degrees C. Frozen liver samples (∼200 mg) were thawed and triglycerides assessed as previously described [28] and quantified using Triglyceride Assay Kit (Sigma).

### Bone Marrow Transplantation

Bone marrow cells were isolated from 129S wildtype and *Npy^/−^* mice and [30] injected IV into lethally irradiated (900 Rad) 6 week old 129S wild-type recipient mice (10 million cells/mouse). Animals were treated with antibiotics (polymyxin and neomycin) for 4 weeks post BM transplantation. Following two weeks of normal chow diet, they were started on ND or HFD diets for 8 weeks. Glucose tolerance testing was performed as described [6] performed after 6 hours of fasting with intraperitoneal injection of 0.7 g/kg of dextrose.

### Statistics

Results are presented as mean ± SEM. Statistical analyses were conducted using an unpaired 2-tailed Student’s *t*-test, with significance set at *p*-value <0.05. Results with multiple groups were first assessed with ANOVA followed by confirmatory analysis, by individual t-test, and bone marrow transplant experiments assessed with two way ANOVA.

## Results

### *Npy* is Induced in SVF Cells in Visceral fat of Obese Mice

**Figure 1:**
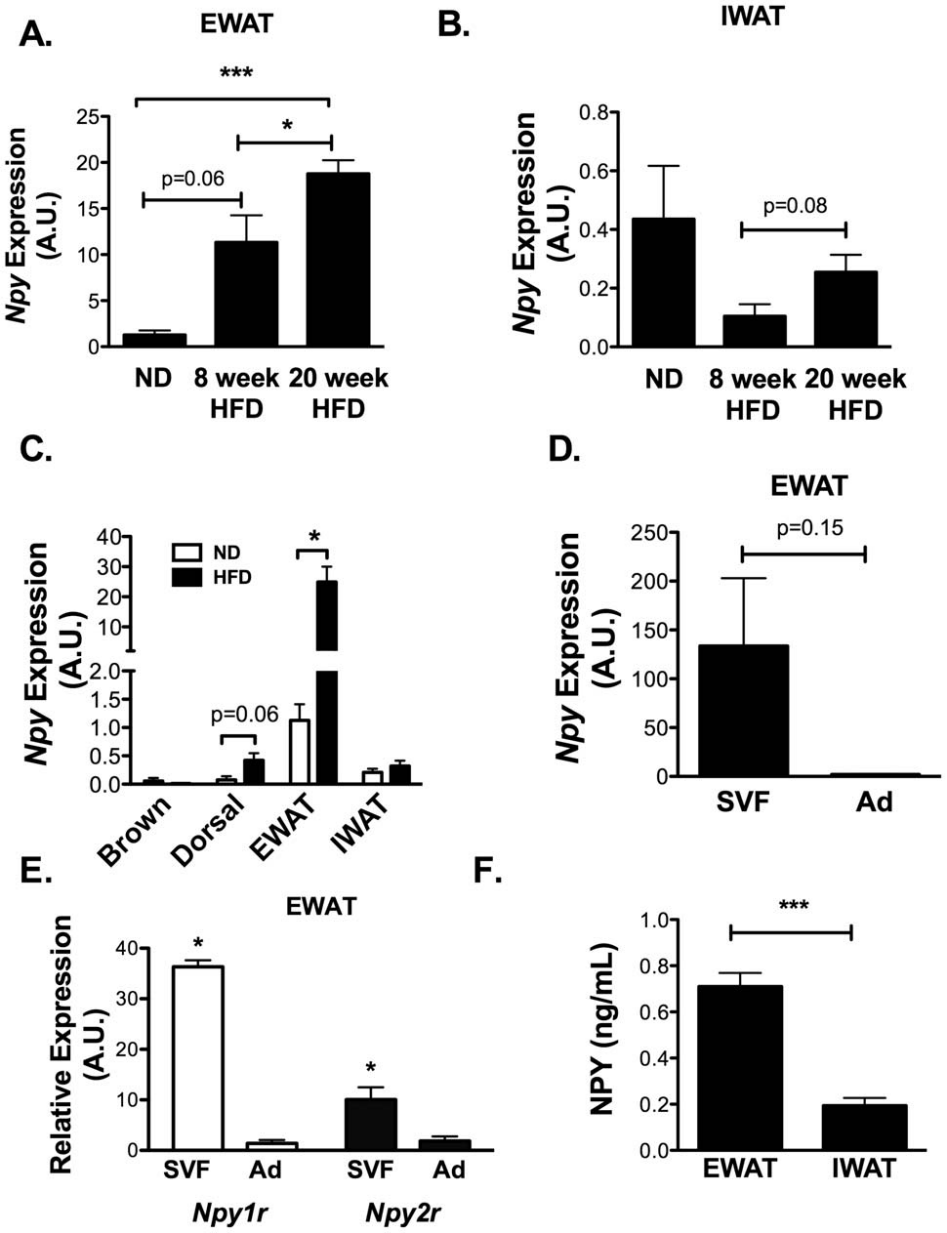
*Npy* induction within visceral fat and SVF with diet-induced obesity. *Npy* gene expression by quantitative RT-PCR in (A) epididymal fat (EWAT) and (B) inguinal fat depots (IWAT) of normal diet (ND), 8 week high fat diet (HFD), and 20 week HFD C57Bl6 mice. N = 4 mice per group. (C) *Npy* expression in various fat depots of ND and 16 week HFD fed animals. N = 4 mice per group. (D) *Npy* expression in stratified adipose tissue. EWAT from HFD C57Bl6 mice was separated into adipocyte (Ad) and stromal vascular fraction (SVF) cells after collagenase digestion. *Npy* expression was assessed by quantitative RT-PCR. N = 4 per group. (E) *Npy1r* and *Npy2r* expression in adipocytes and SVF cell fractions from lean mice. N = 4 per group. (F) NPY protein expression in cultured SVF cells from HFD C57Bl6 mouse EWAT and IWAT. Media was removed from cells after 18 hours of culture and assessed for NPY by ELISA (N = 3 wells per group). *p<0.05, **p<0.01,***p<0.005 by t-test.

To examine the time course and depot specificity of *Npy* expression in mouse models of diet-induced obesity, we examined *Npy* expression in several fat depots from male C57Bl/6 fed a HFD diet (60% kcal from fat). Compared to ND fed mice, *Npy* expression was increased with HFD in a time dependent manner in visceral/epididymal white adipose tissue depots (EWAT; Figure 1A) with induction observed as early as 8 weeks on diet (p = 0.001 by one-way ANOVA). At all time points examined, the expression of *Npy* in inguinal subcutaneous fat depots (IWAT) was significantly lower than EWAT (Figure 1B). In contrast to EWAT, HFD led to no significant changes in the expression of *Npy* in IWAT over time. No significant changes in *Npy* expression were observed in brown adipose depots (BAT) with HFD (Figure 1C), however dorsal subcutaneous fat pads (DWAT) demonstrated a trend toward *Npy* induction with HFD similar to what was seen with EWAT.

To determine the source of NPY in adipose tissue, we examined *Npy* expression in adipocyte and SVF fractions from HFD EWAT after collagenase digestion. Expression of the Y1 and Y2 receptors (*Npy1r* and *Npy2r*) (Figure 1E) were elevated in the SVF compared to adipocyte fractions in obese mice. *Npy* expression tended to be enriched in the SVF fraction, but did not reach statistical significance (Figure 1D; representative of three independent experiments). *Npy5r* expression was not detectable by RT-PCR in SVF or adipocyte fractions in lean or obese mice. To validate the production and secretion of NPY by SVF cells, NPY protein was assessed in the media from cultures of EWAT and IWAT SVF cells from obese mice and higher NPY levels were detected in EWAT conditioned media compared to IWAT (Figure 1F) consistent with the mRNA expression data. The concentration of NPY produced by the SVF (picomolar range) was in physiologic concentrations based on reports from human studies [31], [32].

### NPY is Produced by ATMs and Bone Marrow Derived Myeloid Cells

**Figure 2:**
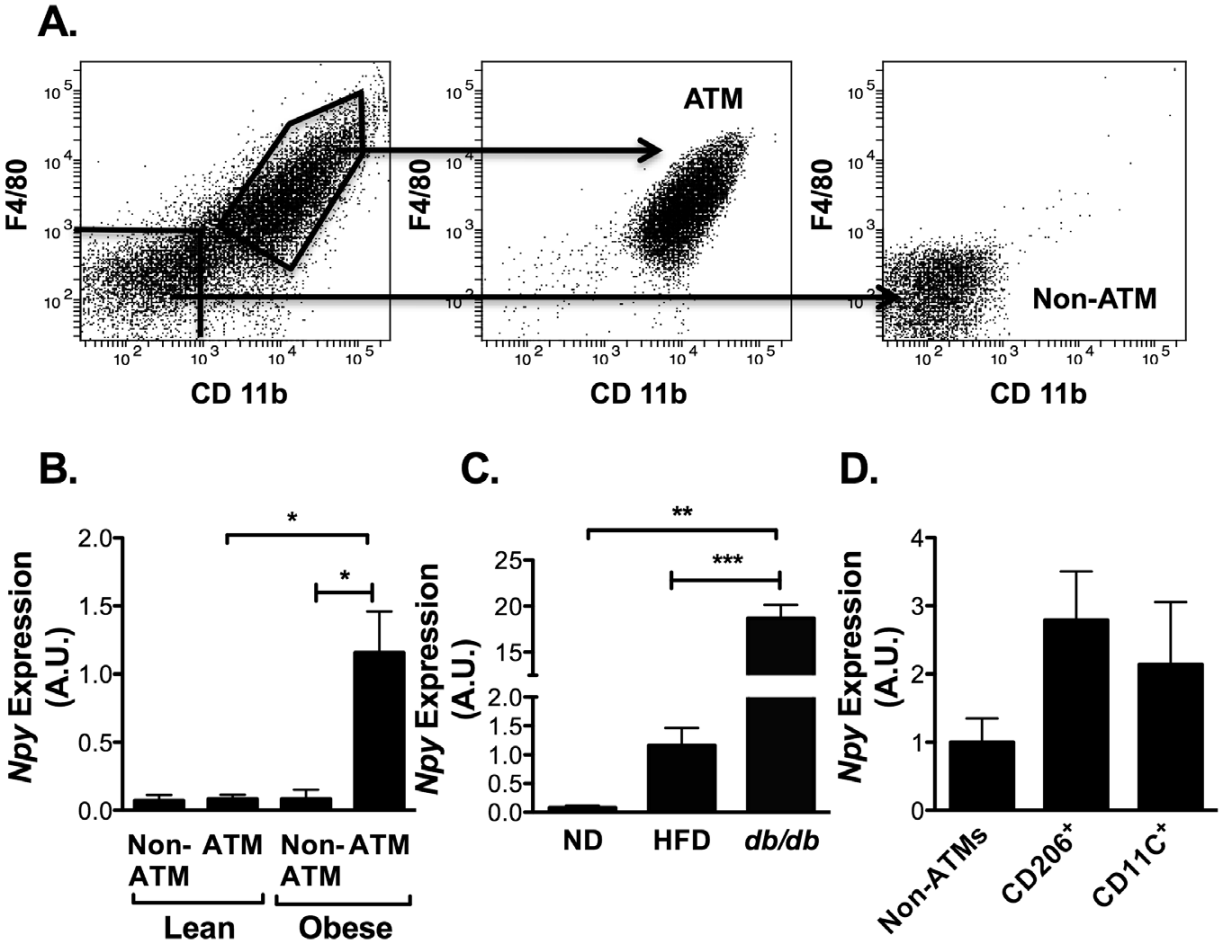
*Npy* gene expression in fat is primarily derived from ATMs in obese animals. (A) Flow cytometry analysis of SVF cells from EWAT of obese C57Bl/6 mice 18–20 wk HFD. SVF cells were stained with F4/80 and CD11b prior to analysis by flow cytometry. Fluorescent activated cell sorting (FACS) isolated purified ATM and non-ATM fractions (middle and right panels show validation plots). (B) *Npy* expression in ATM and non-ATM fractions by quantitative RT-PCR (N = 5 mice per group) (C) Comparative analysis of *Npy* expression in F4/80^+^ CD11b^+^ ATMs from ND, HFD, and *db/db* mice (N = 3 per group). (D) *Npy* expression in FACS purified M2 (CD206^+^) and M1 (CD11c^+^) ATMs (F4/80^+^ CD11b^+^) *p<0.05, **p<0.01,***p<0.005 by t-test.

Sympathetic nerves are a candidate source of NPY in peripheral tissues, however, our SVF preparations do not isolate intact nerves based on visual inspection and immunostaining SVF cells for the axon specific protein GAP-43 (data not shown). This suggested that NPY was produced by a non-neuronal component of the SVF that is induced with obesity. Given the increase in ATM content with obesity and reports of NPY expression in immune cells [33], we evaluated the hypothesis that ATMs are a source of NPY. EWAT SVF from lean and obese mice were separated into ATM (CD11b^+^ F4/80^+^) and non-ATM (CD11b^−^ F4/80^−^) fractions by FACS and followed by RNA isolation (Figure 2A). In lean animals, *Npy* was expressed equally between ATM and non-ATM cells. With HFD, no significant changes were seen in the expression of *Npy* in non-ATM cells, but there was a significant induction of *Npy* in the CD11b^+^ F4/80^+^ ATMs (Figure 2B). A similar induction of *Npy* was seen purified ATMs from *db/db* mice (Figure 2C). This demonstrates that ATMs are a regulated source of *Npy* production and are the primary source of NPY in obese fat. Further cell sorting of M1 (CD206^+^) and M2 ATMs (CD11c^+^) demonstrated similar NPY gene expression in both ATM populations suggesting that *Npy* expression is not a specific feature of one ATM type or another at the gene expression level (Figure 2D).

**Figure 3:**
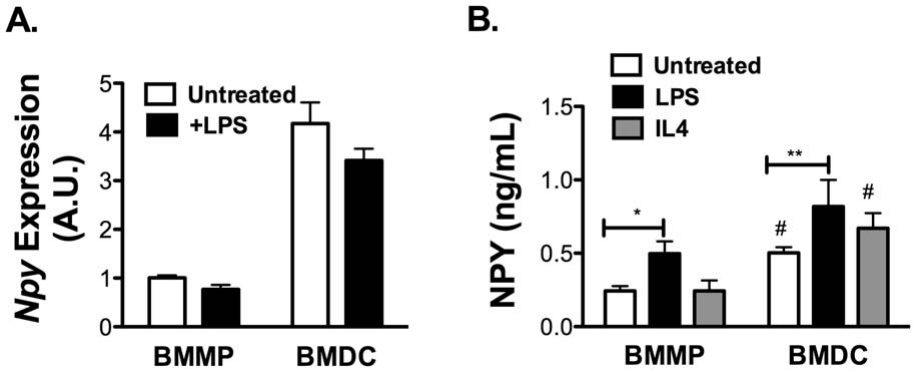
NPY production by bone marrow derived dendritic cells (BMDC) and macrophages (BMMP). (A) Quantitative RT-PCR analysis of *Npy* expression in BMMPs and BMDCs from C57Bl/6 mice with or without LPS (100 ng/ml) for 24 hours. (B) NPY protein expression by ELISA on media from BMDC or BMMP cultured for 24 hours with and without LPS (100 ng/ml) or IL-4 (20 ng/ml) treatment. N = 3 per group. *p<0.05 **p<0.1 #p<0.05 between BMMP and BMDC, by t-test.

Since obesity is known to induce markers of classical M1 activation of ATMs and induce the expression of the dendritic cell (DC) marker CD11c, we examined the influence of inflammatory signals on *Npy* induction in macrophages and DCs. BMMP and BMDCs were treated with M1 (LPS) or M2 (IL-4) skewing stimuli and *Npy* was assessed. *Npy* expression was detectable in both BMMP and BMDC by RT-PCR, however there were no significant changes in *Npy* mRNA expression with either LPS or IL-4 treatment (Figure 3A). However, when the NPY production in the media was quantified, NPY was induced by LPS in the media of both BMMP and BMDC. The specificity of this ELISA assay was confirmed in media from BMDCs derived from NPY KO mice where NPY was below the limit of detection of the assay. Treatment with IL-4 did not significantly alter NPY secretion by either cell type (Figure 3B). In all conditions tested, the mRNA and protein expression of NPY was higher in BMDC than BMMP. These results suggest that the increase in NPY production with obesity is tied to the generation of M1-polarized CD11c^+^ ATMs in obese mice and that the inflammatory induction of NPY is primarily at the level of translational or post-translational regulation.

### NPY 1 Receptors are Present on Non-ATMs and ATMs

**Figure 4:**
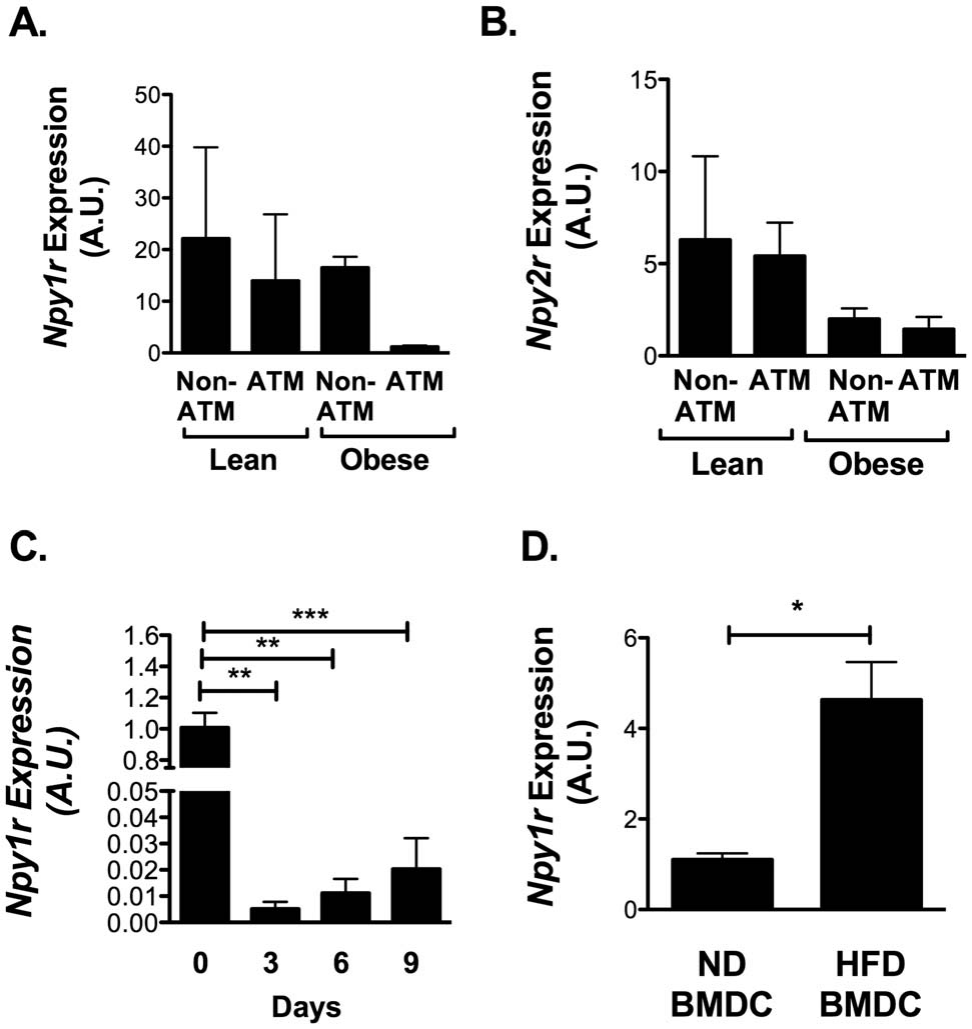
NPY1R is expressed in pre-adipocytes. Analysis of FACS purified ATM and Non-ATM populations from EWAT from 5 lean (ND) and 5 obese (16 wk HFD) C57Bl/6 mice for (A) *Npy1r* and (B) *Npy2r* expression by quantitative RT-PCR. No significant differences between groups by ANOVA. (C) *Npy1r* expression in 3T3-L1 cells during adipocyte differentiation (N = 5). (D) *Npy1r* expression in BMDC from lean and obese (HFD) mice (N = 3 per group) *p<0.05, **p<0.01, ***p<0.005, by t-test.

Fractionation studies of whole adipose tissue demonstrated that NPY receptors *Npy1r* and *Npy2r* are both preferentially expressed in SVF cells relative to the adipocytes in fat (Figure 1E). We next used our FACS sorted SVF populations to examine which cells within adipose tissue are potentially responsive to NPY. *Npy1r and Npy2r* expression was present in both ATMs and non-ATMs at similar levels and was not significantly altered by HFD (Figure 4A and 4B). *Npy5r* expression was not consistently detectable in either adipocyte or SVF fractions (data not shown).

Since the non-ATM population is also enriched for preadipocytes, we examined receptors in these cells by assessing receptor gene expression during the differentiation of 3T3-L1 adipocytes. 3T3-L1 pre-adipocytes had high expression of *Npy1r* that was suppressed during differentiation (Figure 4C). *Npy* mRNA and protein were undetectable at any stage of differentiation of 3T3-L1 pre-adipocytes consistent with our fractionation data (data not shown).

### NPY Antagonists Promote DC Maturation and Pro-inflammatory Gene Expression

While many studies have demonstrated that macrophages and DCs respond to exogenous NPY [15], [24] our initial *in vitro* studies did not show any alterations in macrophage polarization with NPY supplementation (data not shown). We attribute this to the basal production of NPY observed by macrophages and DC. We confirmed the expression of the *Npy1r* in cultured BMDCs and observed an increase in *Npy1r* expression in BMDCs derived from obese mice (Figure 4D). There was very low expression of *Npy2r* and *Npy5r* in these cells by RT-PCR (data not shown). Therefore, an antagonist approach was used to examine the importance of NPY signaling on myeloid cell function.

**Figure 5:**
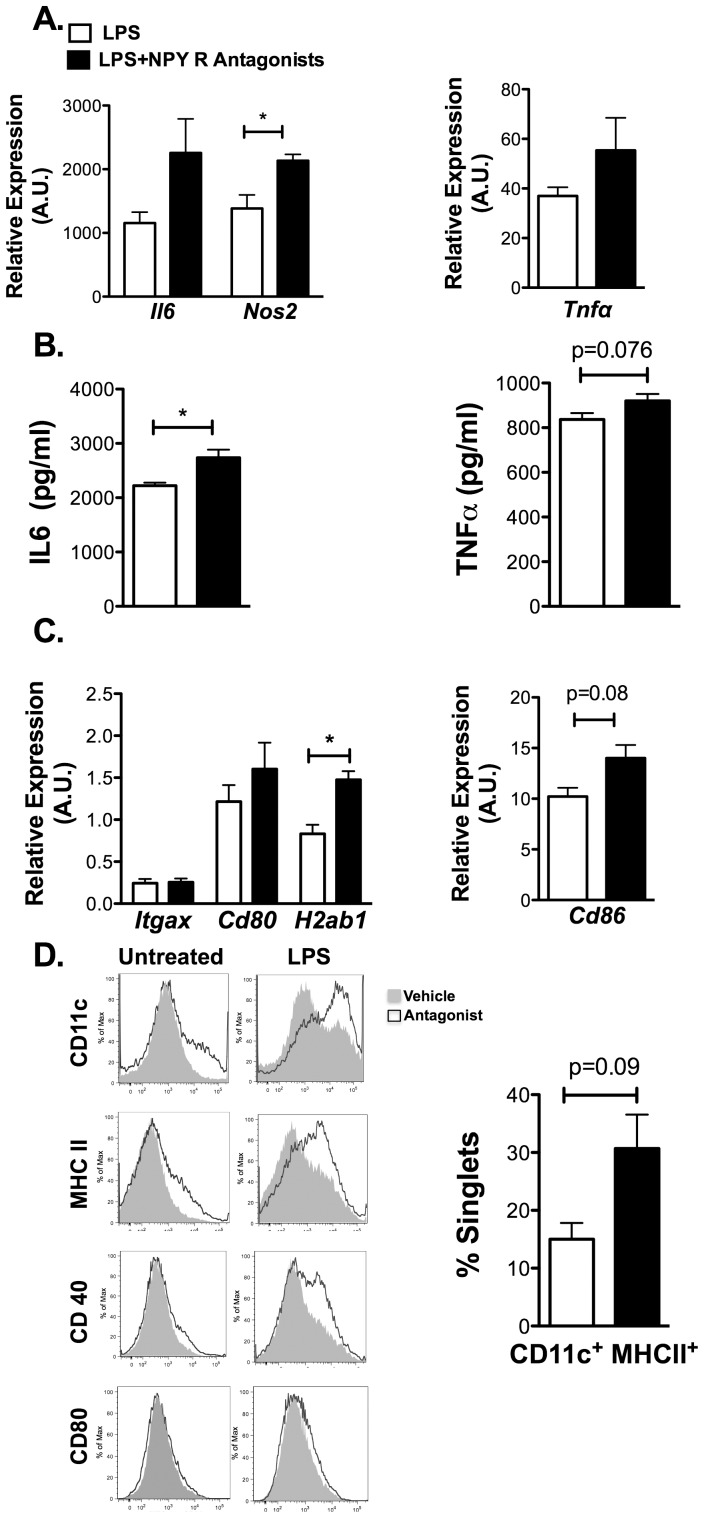
NPY receptor blockade increases M1 cytokine expression and MHCII expression in dendritic cells. Bone marrow dendritic cells (BMDCs) were differentiated for 7 days in the presence of antagonists against the Y1 receptor (BIBO-3304, 10 uM), Y2 receptor (BIIE-0246, 1 uM), and Y5 receptor (L-152,804, 10 uM) and then stimulated with LPS (10 ng/ml) for 18 hours. (A) Analysis of M1 cytokine gene expression by quantitative RT-PCR. (B) TNFα and IL-6 protein in media from cultured BMDC with and without antagonist treatment after stimulation with LPS (10 ng/ml) for 18 hours (N = 6). (C) Analysis of dendritic cell maturation genes in antagonist and vehicle treated BMDCs. (D) Flow cytometry analysis of DC maturation markers in BMDCs with and without antagonist. Right panel shows quantitation of CD11c^+^ MHCII^+^ cells in the BMDC groups. (N = 3). *p<0.05, by t-test.

BMDCs were treated with a cocktail of specific antagonists against Y1, Y2, and Y5 NPY receptors prior to LPS stimulation (M1). Blockade of NPY signaling led to an increase in the expression of proinflammatory genes *Il6, Tnfa,* and *Nos2 (* Figure 5A) and an increase in secreted IL-6 and a trend toward increased TNFα (Figure 5B). While similar trends were seen in unstimulated DCs results were not significant. In addition, DC maturation markers *H2ab1* (MHCII) was increased in NPY receptor antagonist treated DCs compared to vehicle (Figure 5C). Flow cytometry analysis confirmed that combined antagonism of Y1R, Y2R, and Y5R led to an increase in surface maturation markers CD11c^+^, MHCII^+^, and CD40^+^ expression in LPS treated DC with a trend towards an increase in mature CD11c^+^ MHCII^+^ cells (Figure 5D). Overall, these results suggest that NPY has anti-inflammatory effects on DCs that blunt their maturation and inflammatory cytokine production upon LPS stimulation.

### NPY Treatment Decreases Adipose Tissue and Systemic Inflammation in Lean Mice

**Figure 6:**
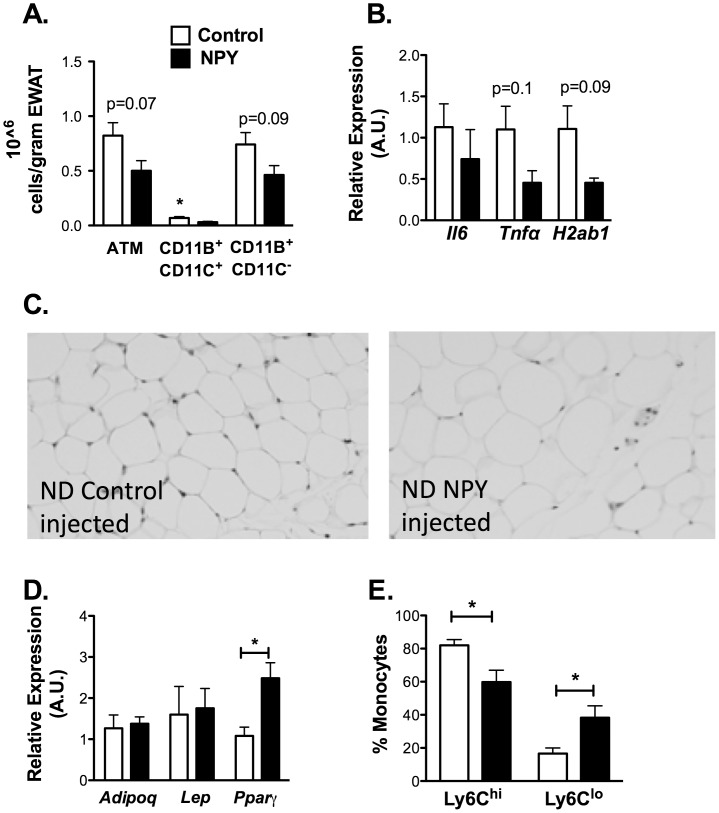
NPY treatment decreases circulating Ly6c^+^ monocyte and CD11c^+^ ATM content. Lean C57Bl/6 mice were treated with NPY or NPY scramble control peptide (60 µg/kg/day) IP for 10 days. N = 4 mice per group. (A) Quantitiation of ATMs by flow cytometry. EWAT SVF cells were stained for ATM markers (F4/80, CD11b, CD11c) and analyzed by flow cytometry. ATM content normalized to fat pad weight. Gene expression analysis of EWAT for (B) inflammatory genes by quantitative RT-PCR. (C) light microscopy image showing larger adipocytes with NPY injection (left panel) compared to scramble control peptide injection (right panel) (D) Gene expression analysis of EWAT for adipocyte genes by quantitative RT-PCR. *p<0.05. (E) Quantitation of Ly6c^hi^ and Ly6c^lo^ CD115^+^ blood monocytes by flow cytometry (N = 4 per group). Data is representative data of one of 3 replicate experiments.

To assess the effects of NPY on adipose tissue inflammation *in vivo*, we sought to simulate the induction of NPY with obesity with NPY injection. NPY or scrambled control peptide were injected I.P. into male C57 mice for 10 days. There were no changes in body weight, EWAT weight, fasting glucose, fasting insulin, liver weight, or liver triglyceride content with NPY treatment (Table S2). The total ATM content in visceral fat showed a trend towards a decrease with NPY treatment (Figure 6A). When ATMs were subdivided based on CD11c expression, NPY injection led to a significant decrease in the quantity of CD11c^+^ ATMs compared to controls and a trend towards a decreased in CD11c^−^ ATMs. Consistent with these changes, the expression of M1 genes *Il6*, *Tnfa*, and *H2ab1* was decreased in NPY injected mice (Figure 6B). NPY injection led to a significant increase in adipocyte size (Figure 6C; Table S2) that correlated with an increase in *Pparg* (p = 0.023) gene expression (Figure 6D). To determine the mechanism of this decrease in CD11c^+^ ATMs, blood monocytes were examined by flow cytometry. While total monocytes were similar in control and NPY injected animals, there was a decrease in classical CD115^+^ Ly6c^hi^ monocytes and an increase in non-classical CD115^+^ Ly6c^lo^ monocytes (Figure 6E). In sum, this data suggests that elevated NPY suppresses CD11c^+^ ATM accumulation in fat by altering the quantity of circulating Ly6c^hi^ monocytes.

Given the suppression of CD11c^+^ ATMs with NPY in lean mice, we examined the possibility that exogenous NPY might blunt inflammation in early obesity in mice. Mice were fed a HFD for 4 weeks prior to treatment with a similar regimen of NPY. No significant changes in body weight, fasting glucose levels, or glucose tolerance measured by GTT were detected in NPY injected mice (Figure S1, Table S3). Analysis of fasting insulin levels demonstrated a trend towards higher insulin levels in the NPY treated group compared to controls (p = 0.09; Table S3). NPY injection induced an enlargement of adipocyte cell size in the HFD mice similar to what was observed in ND fed mice. In contrast with the lean mice, NPY treatment of obese mice did not alter ATM content in EWAT, inflammatory gene expression in fat, or blood monocytes (Figure S1 B-D).

### Lack of NPY in Hematopoietic Cells Potentiates Obesity-induced Inflammation in Adipose Tissue

**Figure 7:**
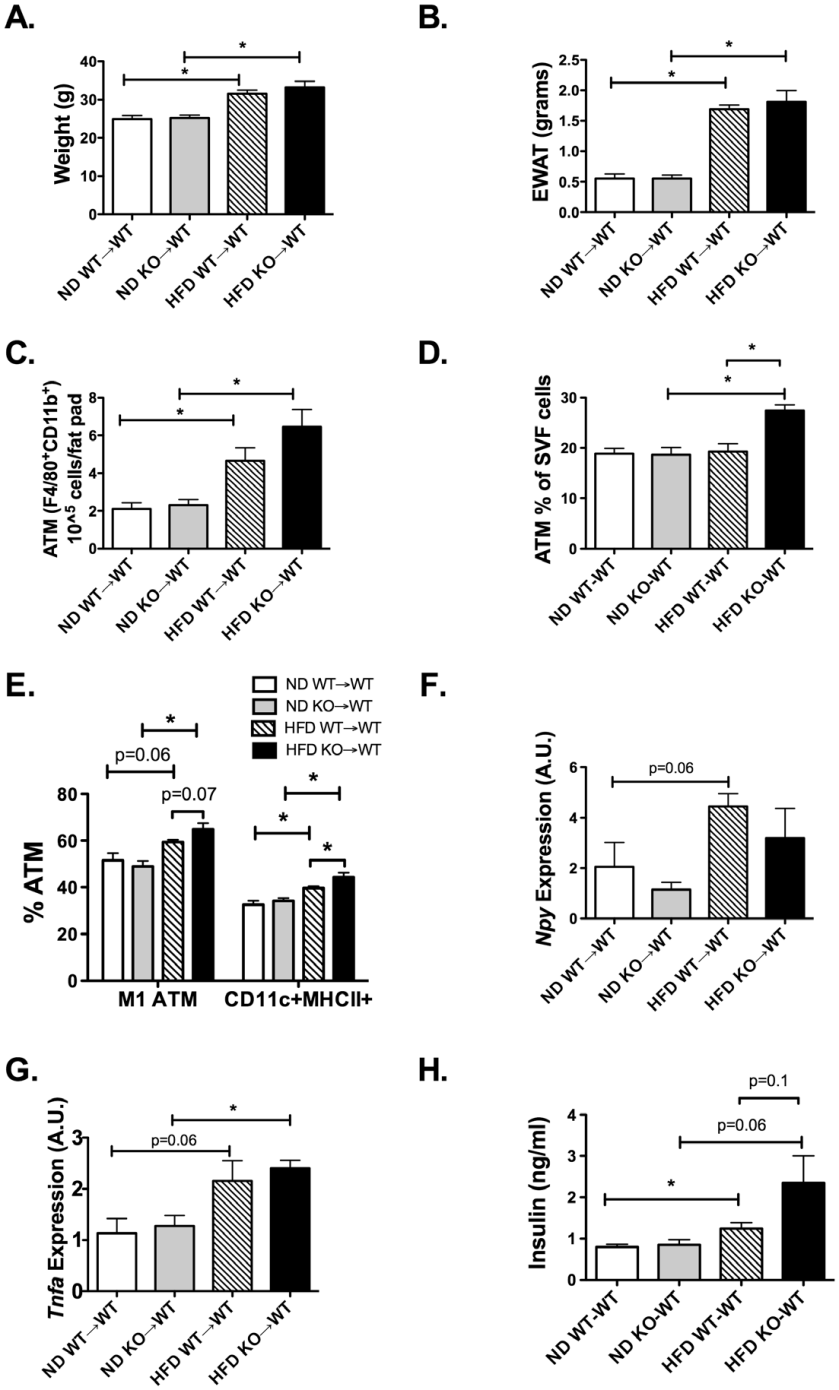
Lack of NPY expression in hematopoietic cells increases M1 ATMs with diet-induced obesity. Donor marrow from S129 wildtype and S129 *Npy^−/−^* mice were transplanted into lethally irradiated wild-type S129 mice. After reconstitution, both groups were placed on ND or HFD chow for 8 weeks (N = 5 in WT donor groups, N = 4 in KO donor groups). (A) Body Weight and (B) EWAT weight assessed at the end of the diet exposure. (C) Flow cytometry quantitation of ATMs (F4/80, CD11b) expressed as number for total per EWAT fat pad (D) ATM as percent of leukocytes in EWAT by flow cytometry. (E) Flow cytometry quantitation of F4/80^+^ CD11b^+^CD11c^+^ ATMs (M1) and F4/80^+^ CD11b^+^CD11c^+^ MHCII^+^ ATMs. (F) *Npy* expression in EWAT of BM chimeras by RT-PCR. (G) *Tnfa* EWAT gene expression by RT-PCR. (H) Fasting insulin levels from serum after 6 hour fast. *p<0.05, groups compared by two-way ANOVA and signficance values shown by individual t-tests.

To examine the significance of NPY production by macrophages/myeloid cells in obesity, we used BM chimeras to examine the hypothesis that loss of NPY from hematopoietic cells would increase adipose tissue inflammation. BM chimeras were generated by injecting donor BM cells from NPY KO or wildtype (WT) mice into lethally irradiated WT recipients. 6 weeks after transplantation, mice were fed either a ND or HFD for 8 weeks. HFD exposure led to significant weight gain and expansion of EWAT weight in mice reconstituted with NPY KO (KO→WT) and WT (WT→WT) marrow (Figure 7A and B). No significant differences in weight were observed between donor groups on either ND or HFD. Overall, there were more ATMs per fat pad in HFD animals (Figure 7C). When we looked at the % ATMs within the SVF there were significant differences amongst all groups (two-way ANOVA p = 0.009) with more ATMs in the HFD KO-WT group (Figure 7D). Stratification of ATMs into subtypes based on CD11c expression demonstrated an increase in the quantity of CD11c^+^ M1 and MHCII^+^ CD11c^+^ ATMs with HFD in both groups. However, obese mice that received BM from NPY KO donors had more CD11c^+^ M1 and MHCII^+^ CD11c^+^ ATMs when compared to WT (Figure 7E) similar to our *in vitro* antagonist studies.

*Npy* EWAT expression in the chimeras was evaluated to assess the contribution of hematopoietic compartment to adipose *Npy* expression. WT→WT mice demonstrated an induction of *Npy* in EWAT with HFD. In contrast, no increase in *Npy* expression with HFD was seen in NPY KO donors (KO→WT) by ANOVA (Figure 7F). *Tnfa* expression of EWAT was induced with HFD in both chimera groups to similar levels (Figure 7G). Evaluation of metabolism showed that glucose intolerance was induced in HFD animals compared to ND, but no significant differences in glucose tolerance was observed between KO→WT and WT→WT animals based on GTT (Figure S2). However, there was a trend to higher fasting insulin levels in KO→WT mice fed HFD suggesting that myeloid NPY deficiency increases insulin resistance with dietary obesity (Figure 7H). When fat cell size was analyzed in the EWAT, adipocytes in the HFD NPY KO-WT group were smaller then those in the HFD WT-WT group (p = 0.043, HFD KO-WT 56.5±1.7 um^2^, HFD WT-WT 61.3±1.6 um^2^. This finding is consistent with the increase in adipocyte size observed with NPY injection into lean mice.

## Discussion

In this study, we report the novel finding that the stress hormone NPY is expressed at physiologic levels by adipose tissue macrophages and participates in obesity-induced adipose tissue inflammation. While macrophage NPY expression is induced by inflammatory signals and obesity, our results support an anti-inflammatory effect of NPY signals on macrophage and DC maturation. *In vitro*, NPY receptor blockade on macrophages/dendritic cells promotes M1 cytokine gene expression and DC maturation.Similarly, BMT *in vivo* experiments showed that the loss of *Npy* expression exclusively in hematopoietic cells increased CD11c^+^ ATMs, however, a significant decrease in cytokine production was not observed. The discrepancy in these observations likely relates to the timing of NPY exposure as well as the fact that *in vivo* experiments involve multiple cell lines that can respond and generate NPY (e.g. adipocytes and nerves). *In vivo*, NPY supplementation decreased M1 ATMs in lean mice by suppressing the levels of circulating Ly6C^hi^ monocytes, an unexpected and novel mechanism linking stress hormones and inflammation.

Our observation that myeloid cells within adipose tissue are a regulated source of NPY production is consistent with other reports of NPY expression in non-neuronal cells. Macrophage NPY expression is evident in publicly available gene expression array data [34]. NPY induction has been reported in stimulated immune cells [33], platelets and endothelial cells [35]–[37] and in the lung [38]. Given our observations, it is possible that macrophages in other important metabolic tissues (e.g. liver and brain) may secrete NPY and influence metabolic tissues. Preliminary studies did not identify significant *Npy* gene expression in microglia however, stress or inflammatory stimuli may be required for significant *Npy* induction, as observed in astrocytes [39]. Our studies also suggest that macrophage NPY secretion can be regulated post-transcriptionally, and therefore mRNA expression techniques may be imprecise in certain contexts.

Our data supporting an anti-inflammatory function for NPY in macrophages, support the evidence that NPY has protective functions during chronic stress in the context of anxiolysis [40] and appetite [41]. Our studies show that this effect is primarily mediated by Y1, Y2, and Y5 receptors in macrophages as gene expression analysis failed to detect the expression of the Y4 receptor in macrophages (data not shown). NPY has also been shown to play a protective role in shock, sepsis, and autoimmune conditions [42], [43]. In models of endotoxemia, NPY can block monocyte induction and improve inflammatory measures [23].

Macrophage derived NPY may participate in the connections between stress and obesity. Clinical studies have shown that increased stress plays a role in weight gain and modifies the risk for diabetes, but the specific mechanisms that link stress pathways with insulin resistance remain unclear [12], [44], [45]. Our results show an early induction of NPY in obese adipose tissue which may dampen the early inflammatory response to obesity. With prolonged exposure to HFD, the anti-inflammatory actions of NPY may be overwhelmed by the ongoing accumulation of M1 ATMs. While our results show an anti-inflammatory function of NPY in lean states and mild obesity, previous work suggests a pro-inflammatory role for NPY in response to the combined effects of stress and HFD [21]. Differences in the levels of NPY induction and alterations in NPY receptor number and type may explain this dichotomy. This hypothesis is consistent with recent studies showing differential NPY receptor contributions to leukocyte activation [46]. In addition, our BM chimera experiments demonstrate that ATMs are a source of NPY in fat, but may not be the sole source. Understanding how factors such as NPY may influence local inflammation is key given that macrophage content and activity within adipose tissue is an important factor for insulin resistance and risk for type 2 diabetes.

Our data support a model where NPY generated from ATMs has autocrine, paracrine, and systemic effects. NPY production by macrophages is increased by HFD and dampens M1 ATM activity. The NPY produced from ATMs may also activate NPY1Rs on pre-adipocytes and adipocytes contributing to a decrease in lipolysis [17], [18], which may further decrease pro-inflammatory signals. At a systemic level, NPY generated from hypertrophied adipose tissue may try to suppress the production/trafficking of circulating classical Ly6c^hi^ monocytes.

While we have observed an induction of CD11c+ ATMs with short term HFD exposure (Figure 7C-D) we were not able to see a significant alteration in glucose metabolism with loss of *Npy* in hematopoietic cells. This may be because inflammation is only one component of the development of insulin resistance and dominates with long-term high fat diet exposure [47]. Since the metabolic phenotype of whole body NPY deficient mice is relatively mild and relates more to diet intake [48], we did not anticipate a substantial metabolic phenotype in our BMT experiment. In addition, the BM chimeras were generated on the 129SV background which is less prone to weight gain with high fat diet and relatively protected from insulin resistance [49].

The end result of NPY signaling is likely complex and highly dependent on the inflammatory microenvironment. Adding to the complexity, NPY can be cleaved by enzymes such as DPPIV (CD26), which is also expressed in macrophages [50], and change the pattern of NPY receptor activation. Our observations may explain why DPPIV inhibitors are capable of improving obesity-induced inflammation [51], [52].

In conclusion, our findings suggest that macrophage derived NPY and its anti-inflammatory effects may serve to preserve normal adipose tissue function in the setting of obesity. Further evaluation of the triggers for this response in adipose tissue may lay the groundwork for novel treatment strategies. Future work is required to fully understand the range of signals that NPY can produce, but this study provides a unique mechanism by which ATMs may influence the adipose tissue environment.

## Supporting Information

Figure S1 **NPY treatment in obese animals does not decrease circulating monocyte or ATM content.** C57Bl6 mice were fed a HFD for 4 weeks prior to treatment with NPY or NPY scramble control peptide (60 µg/kg/day) IP for 10 days. N = 5 per group. (A) Glucose tolerance test. (B) Flow cytometry quantitation of F4/80^+^ CD11b^+^ ATMs. (C) Gene expression analysis of EWAT for inflammatory genes by quantitative RT-PCR. *p<0.05. (D) Quantitation of Ly6c^hi^ and Ly6c^lo^ CD115^+^ blood monocytes by flow cytometry (N = 4 per group), by t-test.
(TIF)

Figure S2 **Lack of NPY expression in hematopoietic cells does not alter glucose tolerance.** Donor marrow from S129 wildtype and S129 *Npy^−/−^* mice were transplanted into lethally irradiated wild-type S129 mice. After reconstitution, both groups were placed on ND or HFD chow for 8 weeks (N = 5 in WT donor groups, N = 4 in KO donor groups). (A) GTT studies in ND animals (B) GTT studies in HFD animals.
(TIF)

Table S1 **Gene primer sequences used for quantitative RT-PCR.**
(DOCX)

Table S2 **Summary of metabolic parameters of lean (ND) mice injected with NPY for 10 days.** N = 4 per group. Data presented ± SEM. *p<0.05 by t-test. Control vs NPY injection
(DOCX)

Table S3 **Summary of metabolic parameters of HFD-fed mice injected with NPY for 10 days.** N = 5 per group. Data presented ± SEM. *p<0.05 by t-test. *p<0.05 Control vs NPY injection
(DOCX)

## References

[1] Berg AH, Scherer PE (2005) Adipose tissue, inflammation, and cardiovascular disease. Circ Res 96: 939–949. 15890981 10.1161/01.RES.0000163635.62927.34

[2] Dedoussis GV, Kapiri A, Samara A, Dimitriadis D, Lambert D, et al (2010) Expression of inflammatory molecules and associations with BMI in children. Eur J Clin Invest 40: 388–392. 20345379 10.1111/j.1365-2362.2010.02277.x

[3] Hotamisligil GS, Arner P, Caro JF, Atkinson RL, Spiegelman BM (1995) Increased adipose tissue expression of tumor necrosis factor-alpha in human obesity and insulin resistance. J Clin Invest 95: 2409–2415. 7738205 10.1172/JCI117936 PMC295872

[4] Lumeng CN, Bodzin JL, Saltiel AR (2007) Obesity induces a phenotypic switch in adipose tissue macrophage polarization. J Clin Invest 117: 175–184. 17200717 10.1172/JCI29881 PMC1716210

[5] Aron-Wisnewsky J, Tordjman J, Poitou C, Darakhshan F, Hugol D, et al (2009) Human adipose tissue macrophages: m1 and m2 cell surface markers in subcutaneous and omental depots and after weight loss. J Clin Endocrinol Metab 94: 4619–4623. 19837929 10.1210/jc.2009-0925

[6] Westcott DJ, Delproposto JB, Geletka LM, Wang T, Singer K, et al (2009) MGL1 promotes adipose tissue inflammation and insulin resistance by regulating 7/4hi monocytes in obesity. J Exp Med 206: 3143–3156. 19995956 10.1084/jem.20091333 PMC2806469

[7] Robbins CS, Chudnovskiy A, Rauch PJ, Figueiredo JL, Iwamoto Y, et al.. (2011) Extramedullary Hematopoiesis Generates Ly-6Chigh Monocytes that Infiltrate Atherosclerotic Lesions. Circulation. 10.1161/CIRCULATIONAHA.111.061986 PMC3263762 22144566

[8] Kosteli A, Sugaru E, Haemmerle G, Martin JF, Lei J, et al (2010) Weight loss and lipolysis promote a dynamic immune response in murine adipose tissue. J Clin Invest 120: 3466–3479. 20877011 10.1172/JCI42845 PMC2947229

[9] Xu H, Barnes GT, Yang Q, Tan G, Yang D, et al (2003) Chronic inflammation in fat plays a crucial role in the development of obesity-related insulin resistance. J Clin Invest 112: 1821–1830. 14679177 10.1172/JCI19451 PMC296998

[10] Goldfine AB, Silver R, Aldhahi W, Cai D, Tatro E, et al (2008) Use of salsalate to target inflammation in the treatment of insulin resistance and type 2 diabetes. Clin Transl Sci 1: 36–43. 19337387 10.1111/j.1752-8062.2008.00026.x PMC2662587

[11] Patsouris D, Li PP, Thapar D, Chapman J, Olefsky JM, et al (2008) Ablation of CD11c-positive cells normalizes insulin sensitivity in obese insulin resistant animals. Cell Metab 8: 301–309. 18840360 10.1016/j.cmet.2008.08.015 PMC2630775

[12] Kyrou I, Chrousos GP, Tsigos C (2006) Stress, visceral obesity, and metabolic complications. Ann N Y Acad Sci 1083: 77–110. 17148735 10.1196/annals.1367.008

[13] Lambert GW, Straznicky NE, Lambert EA, Dixon JB, Schlaich MP (2010) Sympathetic nervous activation in obesity and the metabolic syndrome–causes, consequences and therapeutic implications. Pharmacol Ther 126: 159–172. 20171982 10.1016/j.pharmthera.2010.02.002

[14] Heilig M (2004) The NPY system in stress, anxiety and depression. Neuropeptides 38: 213–224. 15337373 10.1016/j.npep.2004.05.002

[15] Dimitrijevic M, Stanojevic S, Vujic V, Beck-Sickinger A, von Horsten S (2005) Neuropeptide Y and its receptor subtypes specifically modulate rat peritoneal macrophage functions in vitro: counter regulation through Y1 and Y2/5 receptors. Regul Pept 124: 163–172. 15544855 10.1016/j.regpep.2004.07.012

[16] Ruohonen ST, Pesonen U, Moritz N, Kaipio K, Roytta M, et al (2008) Transgenic mice overexpressing neuropeptide Y in noradrenergic neurons: a novel model of increased adiposity and impaired glucose tolerance. Diabetes 57: 1517–1525. 18276767 10.2337/db07-0722

[17] Kos K, Baker AR, Jernas M, Harte AL, Clapham JC, et al (2009) DPP-IV inhibition enhances the antilipolytic action of NPY in human adipose tissue. Diabetes Obes Metab 11: 285–292. 19175376 10.1111/j.1463-1326.2008.00909.x

[18] Yang K, Guan H, Arany E, Hill DJ, Cao X (2008) Neuropeptide Y is produced in visceral adipose tissue and promotes proliferation of adipocyte precursor cells via the Y1 receptor. FASEB J 22: 2452–2464. 18323405 10.1096/fj.07-100735

[19] Segal-Lieberman G, Trombly DJ, Juthani V, Wang X, Maratos-Flier E (2003) NPY ablation in C57BL/6 mice leads to mild obesity and to an impaired refeeding response to fasting. Am J Physiol Endocrinol Metab 284: E1131–1139. 12582011 10.1152/ajpendo.00491.2002

[20] Kuo LE, Czarnecka M, Kitlinska JB, Tilan JU, Kvetnansky R, et al (2008) Chronic stress, combined with a high-fat/high-sugar diet, shifts sympathetic signaling toward neuropeptide Y and leads to obesity and the metabolic syndrome. Ann N Y Acad Sci 1148: 232–237. 19120115 10.1196/annals.1410.035 PMC2914537

[21] Kuo LE, Kitlinska JB, Tilan JU, Li L, Baker SB, et al (2007) Neuropeptide Y acts directly in the periphery on fat tissue and mediates stress-induced obesity and metabolic syndrome. Nat Med 13: 803–811. 17603492 10.1038/nm1611

[22] Bedoui S, Miyake S, Lin Y, Miyamoto K, Oki S, et al (2003) Neuropeptide Y (NPY) suppresses experimental autoimmune encephalomyelitis: NPY1 receptor-specific inhibition of autoreactive Th1 responses in vivo. J Immunol 171: 3451–3458. 14500640 10.4049/jimmunol.171.7.3451

[23] Stadler J, Le TP, Haas P, Nave H (2011) Distinct effects of NPY13–36, a specific NPY Y2 agonist, in a model of rodent endotoxemia on leukocyte subsets and cytokine levels. Annals of anatomy = Anatomischer Anzeiger : official organ of the Anatomische Gesellschaft 193: 486–493. 22074679 10.1016/j.aanat.2011.10.009

[24] Bedoui S, Kuhlmann S, Nave H, Drube J, Pabst R, et al (2001) Differential effects of neuropeptide Y (NPY) on leukocyte subsets in the blood: mobilization of B-1-like B-lymphocytes and activated monocytes. J Neuroimmunol 117: 125–132. 11431012 10.1016/s0165-5728(01)00328-9

[25] Nave H, Bedoui S, Moenter F, Steffens J, Felies M, et al (2004) Reduced tissue immigration of monocytes by neuropeptide Y during endotoxemia is associated with Y2 receptor activation. J Neuroimmunol 155: 1–12. 15342191 10.1016/j.jneuroim.2004.05.009

[26] Wheway J, Herzog H, Mackay F (2007) The Y1 receptor for NPY: a key modulator of the adaptive immune system. Peptides 28: 453–458. 17240480 10.1016/j.peptides.2006.09.030

[27] Gelfo F, De Bartolo P, Tirassa P, Croce N, Caltagirone C, et al (2011) Intraperitoneal injection of neuropeptide Y (NPY) alters neurotrophin rat hypothalamic levels: Implications for NPY potential role in stress-related disorders. Peptides 32: 1320–1323. 21473895 10.1016/j.peptides.2011.03.023

[28] Morris DL, Oatmen KE, Wang T, DelProposto JL, Lumeng CN (2012) CX3CR1 deficiency does not influence trafficking of adipose tissue macrophages in mice with diet-induced obesity. Obesity (Silver Spring) 20: 1189–1199. 22252034 10.1038/oby.2012.7 PMC4006981

[29] Lumeng CN, DelProposto JB, Westcott DJ, Saltiel AR (2008) Phenotypic switching of adipose tissue macrophages with obesity is generated by spatiotemporal differences in macrophage subtypes. Diabetes 57: 3239–3246. 18829989 10.2337/db08-0872 PMC2584129

[30] Erickson JC, Clegg KE, Palmiter RD (1996) Sensitivity to leptin and susceptibility to seizures of mice lacking neuropeptide Y. Nature. 381: 415–421. 10.1038/381415a0 8632796

[31] Kakko T, Jaakkola U, Raitakari OT, Kallio J (2011) Inflammatory effects of blood leukocytes: association with vascular function in neuropeptide Y proline 7-genotyped type 2 diabetes patients. Diab Vasc Dis Res 8: 221–228. 21746772 10.1177/1479164111415882

[32] Abid K, Rochat B, Lassahn PG, Stocklin R, Michalet S, et al (2009) Kinetic study of neuropeptide Y (NPY) proteolysis in blood and identification of NPY3–35: a new peptide generated by plasma kallikrein. J Biol Chem 284: 24715–24724. 19620246 10.1074/jbc.M109.035253 PMC2757175

[33] Schwarz H, Villiger PM, von Kempis J, Lotz M (1994) Neuropeptide Y is an inducible gene in the human immune system. J Neuroimmunol 51: 53–61. 8157736 10.1016/0165-5728(94)90128-7

[34] El Kasmi KC, Smith AM, Williams L, Neale G, Panopoulos AD, et al (2007) Cutting edge: A transcriptional repressor and corepressor induced by the STAT3-regulated anti-inflammatory signaling pathway. J Immunol 179: 7215–7219. 18025162 10.4049/jimmunol.179.11.7215

[35] Chen SH, Han QD (1995) Increase of release of neuropeptide Y in vitro from platelets of spontaneously hypertensive rats. Zhongguo yao li xue bao = Acta pharmacologica Sinica 16: 149–152. 7597917

[36] Myers AK, Farhat MY, Vaz CA, Keiser HR, Zukowska-Grojec Z (1988) Release of immunoreactive-neuropeptide by rat platelets. Biochemical and biophysical research communications 155: 118–122. 3415675 10.1016/s0006-291x(88)81057-x

[37] Silva AP, Cavadas C, Baisse-Agushi B, Spertini O, Brunner HR, et al (2003) NPY, NPY receptors, and DPP IV activity are modulated by LPS, TNF-alpha and IFN-gamma in HUVEC. Regulatory peptides 116: 71–79. 14599717 10.1016/s0167-0115(03)00191-5

[38] Makinde TO, Steininger R, Agrawal DK (2012) NPY and NPY receptors in airway structural and inflammatory cells in allergic asthma. Exp Mol Pathol. 10.1016/j.yexmp.2012.05.009 PMC3488603 22705097

[39] Barnea A, Roberts J, Keller P, Word RA (2001) Interleukin-1beta induces expression of neuropeptide Y in primary astrocyte cultures in a cytokine-specific manner: induction in human but not rat astrocytes. Brain research 896: 137–145. 11277982 10.1016/s0006-8993(01)02141-2

[40] Dallman MF, Pecoraro N, Akana SF, La Fleur SE, Gomez F, et al (2003) Chronic stress and obesity: a new view of “comfort food”. Proc Natl Acad Sci U S A 100: 11696–11701. 12975524 10.1073/pnas.1934666100 PMC208820

[41] Rasmusson AM, Schnurr PP, Zukowska Z, Scioli E, Forman DE (2010) Adaptation to extreme stress: post-traumatic stress disorder, neuropeptide Y and metabolic syndrome. Exp Biol Med (Maywood) 235: 1150–1162. 20881319 10.1258/ebm.2010.009334

[42] Hauser GJ, Dayao EK, Zukowska-Grojec Z (1995) Effect of neuropeptide Y on endotoxin-induced suppression of the response to various agonists in conscious rats. Life Sci 57: 235–244. 7596229 10.1016/0024-3205(95)00266-9

[43] Qureshi NU, Dayao EK, Shirali S, Zukowska-Grojec Z, Hauser GJ (1998) Endogenous neuropeptide Y mediates vasoconstriction during endotoxic and hemorrhagic shock. Regul Pept 75–76: 215–220. 10.1016/s0167-0115(98)00071-8 9802412

[44] Gundersen C, Mahatmya D, Garasky S, Lohman B (2010) Linking psychosocial stressors and childhood obesity. Obes Rev. 10.1111/j.1467-789X.2010.00813.x 21054757

[45] Kivimaki M, Head J, Ferrie JE, Shipley MJ, Brunner E, et al (2006) Work stress, weight gain and weight loss: evidence for bidirectional effects of job strain on body mass index in the Whitehall II study. Int J Obes (Lond) 30: 982–987. 16418750 10.1038/sj.ijo.0803229

[46] Mitic K, Stanojevic S, Kustrimovic N, Vujic V, Dimitrijevic M (2011) Neuropeptide Y modulates functions of inflammatory cells in the rat: distinct role for Y1, Y2 and Y5 receptors. Peptides 32: 1626–1633. 21699939 10.1016/j.peptides.2011.06.007

[47] Lee YS, Li P, Huh JY, Hwang IJ, Lu M, et al (2011) Inflammation is necessary for long-term but not short-term high-fat diet-induced insulin resistance. Diabetes 60: 2474–2483. 21911747 10.2337/db11-0194 PMC3178297

[48] Patel HR, Qi Y, Hawkins EJ, Hileman SM, Elmquist JK, et al (2006) Neuropeptide Y deficiency attenuates responses to fasting and high-fat diet in obesity-prone mice. Diabetes 55: 3091–3098. 17065347 10.2337/db05-0624

[49] Mori MA, Liu M, Bezy O, Almind K, Shapiro H, et al (2010) A systems biology approach identifies inflammatory abnormalities between mouse strains prior to development of metabolic disease. Diabetes 59: 2960–2971. 20713682 10.2337/db10-0367 PMC2963557

[50] Shah Z, Kampfrath T, Deiuliis JA, Zhong J, Pineda C, et al (2011) Long-term dipeptidyl-peptidase 4 inhibition reduces atherosclerosis and inflammation via effects on monocyte recruitment and chemotaxis. Circulation 124: 2338–2349. 22007077 10.1161/CIRCULATIONAHA.111.041418 PMC4224594

[51] Dobrian AD, Ma Q, Lindsay JW, Leone KA, Ma K, et al.. (2010) Dipeptidyl peptidase-4 inhibitor sitagliptin reduces local inflammation in adipose tissue and in pancreatic islets of obese mice. Am J Physiol Endocrinol Metab. 10.1152/ajpendo.00463.2010 PMC3043624 21081706

[52] Kim SJ, Nian C, McIntosh CH (2010) Sitagliptin (MK0431) inhibition of dipeptidyl peptidase IV decreases nonobese diabetic mouse CD4+ T-cell migration through incretin-dependent and -independent pathways. Diabetes 59: 1739–1750. 20368408 10.2337/db09-1618 PMC2889774

