# Correction: Neuropeptide Y Is Produced by Adipose Tissue Macrophages and Regulates Obesity-Induced Inflammation

**DOI:** 10.1371/annotation/c2432ace-1dd7-4b02-8980-e4e6c085beba

**Published:** 2013-09-12

**Authors:** Kanakadurga Singer, David L. Morris, Kelsie E. Oatmen, Tianyi Wang, Jennifer DelProposto, Taleen Mergian, Kae Won Cho, Carey N. Lumeng

Multiple grants and a funding organization were incorrectly omitted from the Funding Statement. The Funding Statement should read: "This work was funded by grants from the National Institutes of Health DK078851, DK090262 (C.N.L.), DK091976 (D.L.M.) and HD007513 (K.S.). Additional support from Endocrine Fellows Foundation and Pediatric Endocrine Society for K.S.. The funders had no role in study design, data collection, decision to publish, or preparation of the manuscript." 

