# Supplementary figures and images for: Neuropeptide Y Is Produced by Adipose Tissue Macrophages and Regulates Obesity-Induced Inflammation
Source: PLoS One. 2013 Mar 5;8(3):e57929. doi: 10.1371/journal.pone.0057929 (PMC3589443; doi:10.1371/journal.pone.0057929)

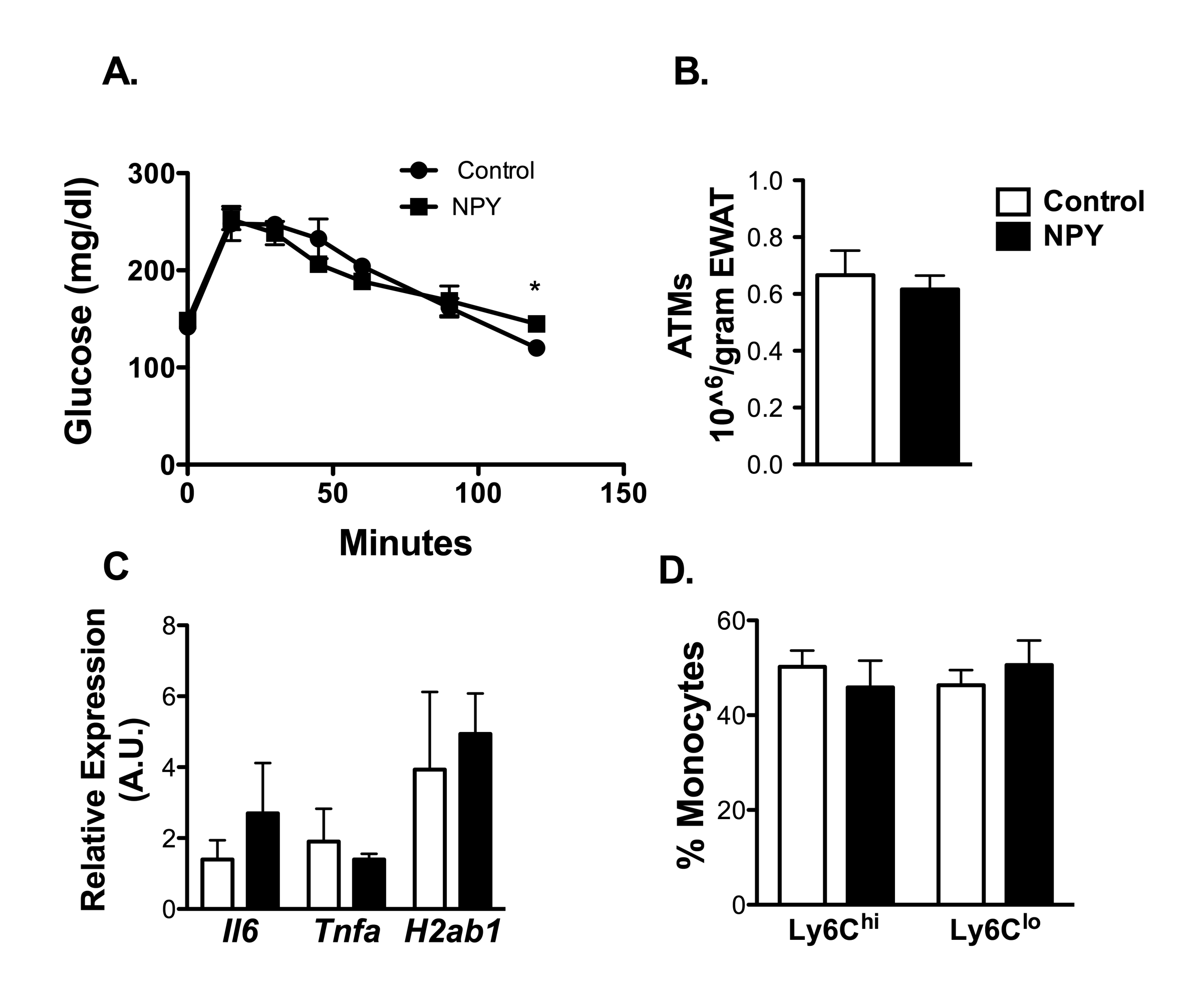

Supplement: Figure S1 — NPY treatment in obese animals does not decrease circulating monocyte or ATM content. C57Bl6 mice were fed a HFD for 4 weeks prior to treatment with NPY or NPY scramble control peptide (60 µg/kg/day) IP for 10 days. N = 5 per group. (A) Glucose tolerance test. (B) Flow cytometry quantitation of F4/80+ CD11b+ ATMs. (C) Gene expression analysis of EWAT for inflammatory genes by quantitative RT-PCR. *p<0.05. (D) Quantitation of Ly6chi and Ly6clo CD115+ blood monocytes by flow cytometry (N = 4 per group), by t-test. (TIF) [file pone.0057929.s001.tif]

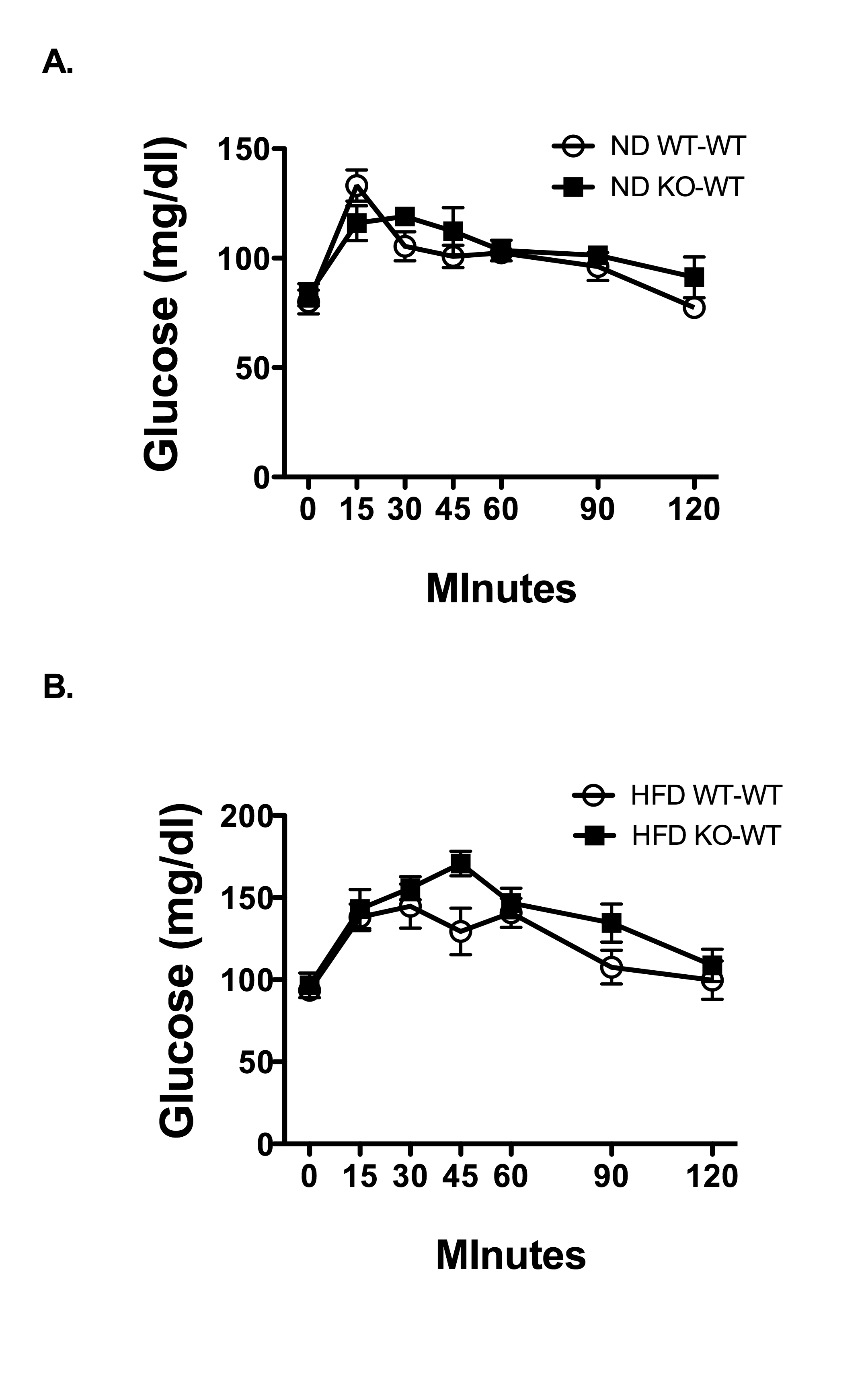

Supplement: Figure S2 — Lack of NPY expression in hematopoietic cells does not alter glucose tolerance. Donor marrow from S129 wildtype and S129 Npy−/− mice were transplanted into lethally irradiated wild-type S129 mice. After reconstitution, both groups were placed on ND or HFD chow for 8 weeks (N = 5 in WT donor groups, N = 4 in KO donor groups). (A) GTT studies in ND animals (B) GTT studies in HFD animals. (TIF) [file pone.0057929.s002.tif]
