# Supplementary material for: Neuropeptide Y Is Produced by Adipose Tissue Macrophages and Regulates Obesity-Induced Inflammation
Source: PLoS One. 2013 Mar 5;8(3):e57929. doi: 10.1371/journal.pone.0057929 (PMC3589443; doi:10.1371/journal.pone.0057929)
Supplement: Table S1 — Gene primer sequences used for quantitative RT-PCR. (DOCX) [file pone.0057929.s003.docx]

| Gene | Forward Primer | Reverse Primer |
| --- | --- | --- |
| *Gapdh* | TGAAGCAGGCATCTGAGGG | CGAAGGTGGAAGAGTGGGAG |
| *Npy* | ATGCTAGGTAACAAGCGAATGG | TGTCGCAGAGCGGAGTAGTAT |
| *Npy1r* | TGATCTCCACCTGCGTCAAC | ATGGCTATGGTCTCGTAGTCAT |
| *Npy2r* | ATCATCTTGCTAGGGGTAGTTGG | CGGACCCATTTTCCACTCTCC |
| *Npy5r* | GTGAACTTTCTCATAGGCAACCT | GCATGATATGGCACATGGCTTT |
| Dpp4 | TCATAGGATCACATCGACAGGAG | AGAAACGTGTTGTTTGGAGACC |
| *Tnfα* | ACGGCATGGATCTCAAAGAC | AGATAGCAAATCGGCTGACG |
| *Il6* | TAGTCC TTCCTACCCCAATTTCC | AAGGAACCCTTAGAGTGCTTACT |
| *Il12* | TGAAGACGG CCAGAGAAAAAC | AAGGAACCCTTAGAGTGCTTACT |
| *Nos2* | CCAAGCCCT CACCTACTTCC | CTCTGAGGGCTGACACAAGG |
| *H2Ab1* | CACTCTGGTCTGTTC GGTGAC | CCTCTCCCTGATGAGGGGTC |
| *Adipo* | GCAGGCATCCCAGGA CATC | GCGATACATATAAGCGGCTTCT |
| *Leptin* | GAGACCCCTGTGTCGGTTC | CTGCGTGTGTGAAATGTCATTG |
| *PPARγ* | TCGCTGATGCACTGCCTATG | GAGAGGTCCACAGAGCTGATT |
