# Supplementary material for: Neuropeptide Y Is Produced by Adipose Tissue Macrophages and Regulates Obesity-Induced Inflammation
Source: PLoS One. 2013 Mar 5;8(3):e57929. doi: 10.1371/journal.pone.0057929 (PMC3589443; doi:10.1371/journal.pone.0057929)
Supplement: Table S2 — Summary of metabolic parameters of lean (ND) mice injected with NPY for 10 days. N = 4 per group. Data presented ± SEM. *p<0.05 by t-test. Control vs NPY injection (DOCX) [file pone.0057929.s004.docx]

|  | ND | ND |
| --- | --- | --- |
|  | Control | NPY |
| Body Weight (g) | 28.9 ± 0.7 | 29.13 ± 1.1 |
| Fasting Insulin (ng/ml) | 0.76 ± 0.08 | 0.80 ± 0.05 |
| Fasting Glucose (mg/dl) | 109.8 ± 9.1 | 103.8 ± 4.1 |
| EWAT Weight (g) | 0.28 ± 0.04 | 0.28 ± 0.03 |
| Adipocyte Size  (Cross-sectional area; µm^2^) | 38.8 ± 1.6 | 54.9 ± 4.1* |
| Liver Weight (g) | 1.42 ± 0.03 | 1.42 ± 0.10 |
| Liver Triglycerides (ug/mg) | 7.95 ± 0.5 | 7.83 ± 0.8 |
