# Supplementary material for: Neuropeptide Y Is Produced by Adipose Tissue Macrophages and Regulates Obesity-Induced Inflammation
Source: PLoS One. 2013 Mar 5;8(3):e57929. doi: 10.1371/journal.pone.0057929 (PMC3589443; doi:10.1371/journal.pone.0057929)
Supplement: Table S3 — Summary of metabolic parameters of HFD-fed mice injected with NPY for 10 days. N = 5 per group. Data presented ± SEM. *p<0.05 by t-test. *p<0.05 Control vs NPY injection (DOCX) [file pone.0057929.s005.docx]

|  | HFD | HFD |
| --- | --- | --- |
|  | Control | NPY |
| Body Weight (g) | 30.3 ± 0.8 | 31.26 ± 1.3 |
| Fasting Insulin (ng/ml) | 1.83 ± 0.5 | 2.90 ± 0.3 |
| Fasting Glucose (mg/dl) | 141.8 ± 7.8 | 148.4 ± 6.9 |
| EWAT Weight (g) | 1.64 ± 0.1 | 1.76 ± 0.2 |
| Adipocyte Size  (Cross-sectional area; µm^2^) | 54.9 ± 1.4 | 61.9 ± 1.4* |
| Liver Weight (g) | 1.01 ± 0.04 | 1.04 ± 0.05 |
| Liver Triglycerides (ug/mg) | 42.9 ± 3.1 | 74.50 ± 10.1* |
